# Breed prevalence of canine lymphoma in South Africa

**DOI:** 10.4102/jsava.v89i0.1530

**Published:** 2018-03-08

**Authors:** Liesl J. van Rooyen, Emma Hooijberg, Fred Reyers

**Affiliations:** 1 Department of Companion Animal Clinical Studies, University of Pretoria, South Africa; 2 IDEXX Laboratories (Pty) Ltd, Johannesburg, South Africa

## Abstract

Lymphoma is a common haematopoietic neoplasm in dogs. Several breeds have been shown to have a predisposition to lymphoma; however, very little information exists regarding the South African dog population. This study assessed whether any breed had increased odds of developing lymphoma compared with others, and also investigated the effects of age, sex and neutering status on disease prevalence. Two study populations and their corresponding reference populations were studied retrospectively. Odds ratios (ORs) for lymphoma in 49 dog breeds, together with their 95% confidence intervals (CI), were calculated. Age effect was assessed by calculating ORs for different age categories in one of the populations. The chi-square test was used to evaluate differences in the prevalence of the various sex and neutering categories in one lymphoma population compared with its reference population. Fourteen breeds had significantly increased odds of developing lymphoma, and one breed had significantly decreased odds (*p* < 0.050). The median ages of the two lymphoma populations were 6.5 and 8.0 years, with the 6.1–9.0 year category having significantly increased odds of developing lymphoma (OR 1.61, CI 1.2–2.16, *p* = 0.002). In one of the lymphoma populations, higher proportions of males (*p* = 0.033) and neutered females (*p* = 0.006) were found when compared with the reference population. These findings suggest that certain breeds in South Africa have a higher risk of developing lymphoma, and that sex hormones may play a role in lymphoma pathogenesis. The findings may provide useful information for pet owners and veterinarians.

## Introduction

Lymphoma is the most common haematopoietic neoplasm of dogs (Dobson 2013) and is a significant cause of morbidity and mortality in canine patients, accounting for 7% – 24% of all canine neoplasia (Kaiser 1981; Moulton & Harvey 1990). The annual incidence is estimated to be between 13 and 24 cases per 100 000 dogs at risk (Backgren 1965; Dorn et al. 1968). This disease is characterised by a clonal expansion of malignant lymphocytes which may occur either in the primary and secondary lymphoid tissues (Lobetti 2009) or within any organ owing to the continuous trafficking of lymphocytes (Vail & Young 2007). The disease typically affects middle-aged to older dogs (Backgren 1965; Jacobs, Messick & Valli 2002) and is frequently characterised by generalised peripheral lymphadenomegaly (Fournel-Fleury et al. 2002; Greenlee et al. 1990; Ponce et al. 2010). Various non-specific clinical signs may also be noted, which include weight loss, lethargy, anorexia and gastrointestinal signs (Fournel-Fleury et al. 2002; Greenlee et al. 1990). Additional clinical findings such as hepatosplenomegaly, the presence of a mediastinal mass and polyuria and/or polydipsia may be observed, depending on the sites that are involved and the subtype of disease (Fournel-Fleury et al. 2002). Histologically, various morphological subtypes with differing biological behaviour have been shown to exist in humans and dogs (Valli et al. 2013).

Despite the importance of this disease, the causative factors are somewhat poorly defined (Lobetti 2009; Modiano et al. 2005; Pastor et al. 2009; Vail & Young 2007). In humans, environmental agents, genetic predispositions, immune dysregulation and various infectious agents have been postulated as potential aetiologies (Cartwright et al. 1999). A similar situation is thought to exist for dogs (Pastor et al. 2009; Vail & Young 2007), although no viral causes have been definitively identified (Fournel-Fleury et al. 2002; Pastor et al. 2009; Teske et al. 1994). In addition to various potential environmental causative agents such as paints, solvents (Gavazza et al. 2001) and herbicides (Hayes et al. 1991), a number of genetic abnormalities have been implicated in the pathogenesis of lymphoma. These include chromosomal aberrations (Modiano et al. 2005), somatic and germline mutations (Veldhoen et al. 1998) and epigenetic modifications (Pelham, Irwin & Kay 2003). Various case series have documented the clustering of lymphoma within groups of related dogs (Lobetti 2009; Onions 1984; Teske et al. 1994), which lends support to the role of genetic factors, of which some have been described previously (Stone et al. 1991a; Stone, Jacky & Prieur 1991b). Furthermore, it has been shown that certain dog breeds, such as the Boxer, have susceptibility or a breed predisposition to lymphoma (Edwards et al. 2003; Priester 1967; Teske 1994).

While various epidemiological studies on breed prevalence of canine lymphoma have been conducted in a number of geographic locations in Europe (Blackwood, Sullivan & Lawson 1997; Cora et al. 2016; Dobson & Gorman 1993; Edwards et al. 2003; Ernst et al. 2016; Gruentzig et al. 2015; Jagielski et al. 2002; Merlo et al. 2008; Pastor et al. 2009; Sapierzynski et al. 2010; Teske 1994), North America (Dorn, Taylor & Hibbard 1967; Dorn et al. 1968; Keller et al. 1993; Villamil et al. 2009; Zemann et al. 1998) and South America (Neuwald et al. 2014), very little information exists regarding the situation in South Africa. Observations by one of the authors (F.R.) suggest that certain popular breeds such as the South African Boerboel, Rhodesian Ridgeback, Staffordshire Bull Terrier and Labrador Retriever appear to be predisposed. However, such observations are confounded by the fact that these breeds are popular with the owner population, and a higher number will be presented for veterinary care for this condition.

It was hypothesised that certain breeds in South Africa would have a higher prevalence and higher risk of lymphoma compared with the general canine population. This study assessed whether certain dog breeds had greater odds of developing lymphoma compared with other breeds using an appropriate reference population to serve as an estimate for the local canine population. Knowledge of such breed predispositions could provide useful information for practicing veterinarians, breeders and pet insurance companies. An additional aim of this study was to investigate the effect of age, sex and neutering status on the prevalence of lymphoma by determining whether dogs in different age categories had greater odds of developing the disease compared with others, and whether the prevalence of lymphoma in certain sex or neutering groups was higher than the prevalence of the sex group in the relevant reference population.

## Research method and design

### Study design and inclusion criteria

This study was a retrospective data analysis. The study was conducted using laboratory data generated by IDEXX Laboratories South Africa during the period from 01 March 2014 to 31 July 2016 (known as the IDEXX population), and the Section Clinical Pathology of the Faculty of Veterinary Science of the University of Pretoria (known as the CP population) during the period 01 January 2014–30 June 2016.

Both databases were searched using the term ‘lymphoma’. Cytological reports containing these search terms were retrieved and reviewed. Cases were included in the study population on the basis of a cytological diagnosis of lymphoma (see below), and if the breed was known. Where available, histopathology and clinical findings were incorporated to strengthen the diagnosis. In instances where an equivocal diagnosis had initially been made, the slides were reviewed by either a board-certified clinical pathologist (E.H.) or a clinical pathology resident (L.J.v.R.) before being included in the study.

Specifically, a cytological diagnosis of lymphoma was made if a sample obtained from lymphoid tissue (lymph nodes, spleen) displayed a monomorphic population (> 50%) of immature lymphoid cells (Messick 2014). Cells were classified as immature if they displayed the following characteristics (Munasinghe et al. 2015; Sozmen et al. 2005): increased cell size (nucleus > 1.5 × the size of a red blood cell), multiple abnormal nucleoli, finely stippled or coarse chromatin and nuclear shape atypia. Additional characteristics included increased cytoplasmic basophilia and abnormal mitoses (Munasinghe et al. 2015; Sozmen et al. 2005). Similarly, a diagnosis of lymphoma was made if there was evidence of organ infiltration (e.g. liver, kidney) by immature-appearing lymphoid cells, or if a body cavity effusion contained low to high numbers of a monomorphic population of immature lymphocytes.

In addition, the clinical records of the cases seen by the Section Clinical Pathology, and the database of the Anatomical Pathology Section of IDEXX Laboratories South Africa, were searched to identify which cases were confirmed by means of histopathology or immunophenotyping.

Cases were excluded in the following instances: if no information regarding the breed was available, if the cytological findings were not consistent with the above-mentioned criteria or if the histopathological findings (where available) yielded a diagnosis other than lymphoma. All included cases were captured into a spreadsheet, and the following data were recorded: breed, age, sex, neutering status and cytological diagnosis.

Three reference populations were created for the purpose of comparison. The first of these was a breakdown according to breed of all of the canine patients for which samples had been accessioned by the clinical pathology laboratory during the study period. A similar breed list was compiled for all of the canine cases that were accessioned by the Johannesburg branch of IDEXX Laboratories South Africa during the study period; this particular breed list also contained information about age and neutering status of individuals (where available). Data from a pet insurance company were also obtained in order to provide an indication of breed prevalence in a non-laboratory population. This contained the following information: total number of insured dogs on the database, as well as a breakdown of this number according to breed.

### Statistical analysis

Each population was evaluated separately and was compared to the reference population generated for the particular laboratory in question. Firstly, the prevalence of lymphoma in each study population was calculated by dividing the total number of cases with lymphoma by the total number of dogs in the relevant reference population. Similarly, the frequency of each breed and prevalence of lymphoma in each breed in each population was calculated. The prevalence of lymphoma in each study population was compared using a chi-square (*χ*^2^) test. When the information was available, the prevalence of each sex and neutering category was calculated. A *χ*^2^ test was used to assess for differences in sex and neutering status between the IDEXX lymphoma population and the IDEXX reference population, and between the two lymphoma study populations. The median age for each lymphoma population was calculated; in the case of the IDEXX lymphoma population, this was compared with the median age of the IDEXX reference population by means of a Mann–Whitney *U* test. The median age for the two lymphoma populations were also compared using the same statistical methods. Data on age, sex and neutering status were not available for the CP reference population.

To assess for possible breed predispositions, the odds ratio (OR) with 95% confidence intervals (CIs) was calculated for each breed. The OR is the odds of a dog of a particular breed developing lymphoma compared with all other breeds in a particular reference population (Szumilas 2010). In this context, an OR > 1.00 indicates possible increased risk of developing lymphoma, whereas an OR < 1.00 suggests a decreased risk. The CI is a measure of uncertainty of the true value of the OR which takes the sample size into account. The narrower the CI, the more precise the OR; and the CI should not contain the value 1.00 for the OR to be considered significant (Szumilas 2010). The CI and thus imprecision of the OR increase when very small sample sizes are used. The OR was therefore not calculated for breeds where the number of animals in either reference population was less than five.

To further explore the influence of age, the IDEXX reference and lymphoma populations were stratified into the following age categories: 0.0–3.0 years, 3.1–6.0 years, 6.1–9.0 years, 9.1–12.0 years, 12.1–15.0 years, 15.1–18.0 years and 18.1 years and above. Once cases had been grouped into these categories, the OR of each age category for developing lymphoma compared with all other categories was calculated. In addition, 95% CIs were also calculated for each OR.

A *p*-value of < 0.050 was considered statistically significant.

All statistical analyses were performed using Microsoft Excel (Microsoft Corp., Redmond, WA, USA) and MedCalc Statistical Software version 16.4.3 (MedCalc Software bvba, Ostend, Belgium).

## Results

Table 1 shows a comparison of the two laboratory reference populations in terms of breed frequency. Breed information from the insurance database is also included in Table 1, for reference. The breeds in which lymphoma was identified during the study period, as well as in previous studies, are listed individually in the table. In both the IDEXX and the CP populations, these breeds made up over 90% of the reference populations, and over 80% of the insurance population.

**TABLE 1:** List of dog breeds, as well as their absolute numbers and frequencies, in each reference population as well as in a pet insurance population.

Breed	IDEXX reference population		Section Clinical Pathology reference population		Insurance database	
	Number	Frequency (%)	Number	Frequency (%)	Number	Frequency (%)
Jack Russell Terrier	2314	8.15	761	13.66	1534	5.20
Dachshund	2213	7.79	540	9.70	2198	7.45
Yorkshire Terrier	1587	5.59	369	6.63	1625	5.51
Labrador Retriever	2289	8.06	339	6.09	1864	6.32
Boerboel	1001	3.52	267	4.79	1148	3.89
German Shepherd	1792	6.31	195	3.50	2311	7.83
Rottweiler	674	2.37	168	3.02	1298	4.40
Mixed Breed	1303	4.59	163	2.93	246	0.83
Maltese Poodle	1237	4.36	161	2.89	1365	4.63
Fox Terrier	506	1.78	142	2.55	352	1.19
American Pit Bull Terrier	324	1.14	134	2.41	480	1.63
Doberman Pinscher	271	0.95	130	2.33	360	1.22
Cocker Spaniel	445	1.57	113	2.03	604	2.05
Bull Terrier	528	1.86	110	1.98	657	2.23
Pomeranian	435	1.53	108	1.94	334	1.13
Border Collie	504	1.77	96	1.72	498	1.69
Great Dane	393	1.38	89	1.60	568	1.92
Beagle	404	1.42	88	1.58	280	0.95
Husky	600	2.11	87	1.56	617	2.09
Staffordshire Bull Terrier	551	1.94	87	1.56	335	1.14
Schnauzer	667	2.35	81	1.45	344	1.17
Boxer	370	1.30	75	1.35	583	1.98
Chihuahua	289	1.02	75	1.35	234	0.79
Miniature Pinscher	453	1.59	75	1.35	63	0.21
Scottish Terrier	486	1.71	73	1.31	127	0.43
English Bulldog	397	1.40	71	1.27	990	3.35
Rhodesian Ridgeback	456	1.61	71	1.27	439	1.49
Golden Retriever	770	2.71	60	1.08	724	2.45
Basset Hound	331	1.17	59	1.06	503	1.70
Boston Terrier	228	0.80	58	1.04	314	1.06
Poodle	392	1.38	47	0.84	504	1.71
Shar-Pei	196	0.69	27	0.48	416	1.41
Belgian Shepherd	58	0.20	20	0.36	121	0.41
Bouvier des Flandres	101	0.36	19	0.34	15	0.05
Newfoundland	39	0.14	18	0.32	46	0.16
Bullmastiff	129	0.45	14	0.25	32	0.11
Greyhound	65	0.23	9	0.16	8	0.03
Pointer (Unspecified)	76	0.27	7	0.13	5	0.02
Whippet	45	0.16	5	0.09	50	0.17
Australian Shepherd	33	0.12	4	0.07	12	0.04
Springer Spaniel	41	0.14	3	0.05	66	0.22
Black Russian Terrier	27	0.10	2	0.04	17	0.06
Mastiff	11	0.04	2	0.04	3	0.01
Hungarian Vizsla	22	0.08	1	0.02	50	0.17
Kerry Blue Terrier	13	0.05	1	0.02	4	0.01
Schipperke	12	0.04	1	0.02	21	0.07
Chow Chow	219	0.77	0	0.00	303	1.03
Spaniel (Unspecified)	291	1.02	0	0.00	0	0.00
Terrier (Unspecified)	213	0.75	0	0.00	32	0.11
Airedale Terrier	48	0.17	4	0.07	19	0.06
American Staffordshire Terrier	33	0.12	32	0.57	955	3.24
Bernese Mountain Dog	16	0.06	2	0.04	17	0.06
Briard	0	0.00	0	0.00	3	0.01
Collie	129	0.45	6	0.11	65	0.22
Corgi	91	0.32	11	0.20	95	0.32
Gordon Setter	1	0.00	0	0.00	0	0.00
Irish Setter	32	0.11	2	0.04	33	0.11
Irish Water Spaniel	0	0.00	0	0.00	0	0.00
Irish Wolfhound	126	0.44	4	0.07	46	0.16
Old English Sheepdog	6	0.02	0	0.00	2	0.01
Saint Bernard	91	0.32	22	0.40	176	0.60
Other breeds	2149	7.53	461	8.28	3401	11.52
Total number of dogs	28 523	100.00	5569	100.00	29 512	100.00

Breeds in which lymphoma was identified in this and previous studies are listed individually.

### IDEXX Laboratories database

A total of 304 dogs were identified from the initial database search, of which 91 were excluded based on the aforementioned criteria. Thus, 213 dogs with lymphoma were included from the IDEXX database during the study period. The reference population consisted of 28 523 dogs for which samples had been accessioned during the same period. The prevalence of lymphoma in the IDEXX study population was 7.5 per 1000 dogs (0.75%).

Table 2 shows the prevalence of lymphoma in each breed as well as the OR of the different dog breeds for developing lymphoma (only when more than five individuals are represented). Fourteen dog breeds were found to have either significantly increased OR (*n* = 13) or decreased OR (*n* = 1).

**TABLE 2:** Prevalence, odds ratios and their confidence intervals for lymphoma in 49 dog breeds from two study populations.

Breed	IDEXX population				Section of Clinical Pathology population			
	Absolute number	Prevalence of lymphoma (%)	Odds ratio	95% confidence interval	Absolute number	Prevalence of lymphoma (%)	Odds ratio	95% confidence interval
Jack Russell Terrier	11	0.48	0.62	0.33–1.14	3	0.39	0.53	0.16–1.72
Dachshund	9	0.41	0.53	0.27–1.04	1	0.19	0.25	0.03–1.80
Yorkshire Terrier	1	0.06	0.08	0.01–0.57	1	0.27	0.37	0.05–2.72
Labrador Retriever	29	1.27	1.83*	1.24–2.72	5	1.47	2.30	0.90–5.93
Boerboel	21	2.10	3.07*	1.95–4.85	2	0.75	1.08	0.26–4.51
German Shepherd	8	0.45	0.58	0.29–1.19	2	1.03	1.51	0.36–6.29
Rottweiler	9	1.34	1.85	0.94–3.62	1	0.60	0.85	0.11–6.24
Mixed Breed	11	0.84	1.15	0.62–2.11	1	0.61	0.88	0.12–6.44
Maltese Poodle	2	0.16	0.21*	0.05–0.84	0	0.00	-	-
Fox Terrier	1	0.20	0.26	0.04–1.87	2	1.41	2.10	0.50–8.78
American Pit Bull Terrier	2	0.62	0.83	0.21–3.36	2	1.49	2.22	0.53–9.33
Doberman Pinscher	2	0.74	1.00	0.25–4.03	1	0.77	1.11	0.15–8.15
Cocker Spaniel	2	0.45	0.60	0.15–2.43	2	1.77	2.66	0.63–11.17
Bull Terrier	8	1.52	2.1*	1.03–4.28	1	0.91	1.32	0.18–9.69
Pomeranian	1	0.23	0.31	0.04–2.18	0	0.00	-	-
Border Collie	9	1.79	2.5*	1.27–4.90	2	2.08	3.15	0.75–13.25
Great Dane	3	0.76	1.03	0.33–3.24	0	0.00	-	-
Beagle	3	0.74	1.00	0.32–3.14	0	0.00	-	-
Husky	3	0.50	0.67	0.21–2.09	2	2.30	3.49	0.83–14.70
Staffordshire Bull Terrier	5	0.91	1.23	0.51–3.00	1	1.15	1.68	0.23–12.36
Schnauzer	4	0.60	0.80	0.30–2.17	1	1.23	1.81	0.25–13.31
Boxer	8	2.16	3.04*	1.49–6.2	0	0.00	-	-
Chihuahua	1	0.35	0.46	0.06–3.31	2	2.67	4.07	0.97–17.20
Miniature Pinscher	1	0.22	0.29	0.04–2.09	0	0.00	-	-
Scottish Terrier	1	0.21	0.27	0.04–1.95	1	1.37	2.01	0.27–14.83
English Bulldog	8	2.02	2.82*	1.38–5.76	0	0.00	-	-
Rhodesian Ridgeback	10	2.19	3.10*	1.63–5.89	1	1.41	2.07	0.28–15.27
Golden Retriever	6	0.78	1.05	0.47–2.38	1	1.67	2.46	0.33–18.20
Basset Hound	6	1.81	2.51*	1.11–5.70	1	1.69	2.50	0.34–18.52
Boston Terrier	1	0.44	0.59	0.08–4.2	0	0.00	-	-
Poodle	2	0.51	0.68	0.17–2.76	0	0.00	-	-
Shar-Pei	3	1.53	2.10	0.67–6.61	0	0.00	-	-
Belgian Shepherd	3	5.17	7.39*	2.30–23.82	1	5.00	7.69*	1.00–58.89
Bouvier des Flandres	1	0.99	1.34	0.19–9.66	0	0.00	-	-
Newfoundland	0	0.00	-	-	1	5.56	8.59*	1.11–66.22
Bullmastiff	4	3.10	4.35*	1.59–11.88	0	0.00	-	-
Greyhound	1	1.54	2.10	0.29–15.19	0	0.00	-	-
Pointer (Unspecified)	2	2.63	3.64	0.89–14.94	0	0.00	-	-
Whippet	1	2.22	3.05	0.42–22.26	0	0.00	-	-
Australian Shepherd	1	3.03	4.20	0.57–30.88	0	0.00	-	-
Springer Spaniel	0	0.00	-	-	1	33.33	-	-
Black Russian Terrier	1	3.70	5.17	0.70–38.27	0	0.00	-	-
Mastiff	1	9.09	13.45*	1.71–105.5	0	0.00	-	-
Hungarian Vizsla	1	4.55	6.40	0.86–47.82	0	0.00	-	-
Kerry Blue Terrier	1	7.69	11.21*	1.45–86.58	0	0.00	-	-
Schipperke	1	8.33	12.22*	1.57–95.13	0	0.00	-	-
Chow Chow	1	0.46	0.61	0.08–4.39	0	0.00	-	-
Spaniel (Unspecified)	2	0.69	0.93	0.23–3.75	0	0.00	-	-
Terrier (Unspecified)	1	0.47	0.63	0.09–4.51	0	0.00	-	-

Odds ratios are only shown for breeds represented by more than five animals in the reference populations.

* , *p* < 0.050

The median age of dogs with lymphoma in the IDEXX population was 8.0 years (range: 9.0 months – 15.0 years); the median age for the IDEXX reference population was 7.0 years (range: 7.0 days – 20.0 years). The difference between the age distribution of the two groups was found to be significant (*p* = 0.015). When the OR for developing lymphoma for the different age categories were calculated, it was found that dogs in the 6.1–9.0 year category had a significantly higher OR (OR 1.61, CI 1.20–2.16) compared with all other groups (*p* = 0.002), and the 0.0–3.0 year group had a significantly decreased OR (OR 0.26, CI 0.13–0.47) compared with all other groups (*p* < 0.001) (Table 3).

**TABLE 3:** Odds ratios with their confidence intervals for lymphoma in the different age categories of the IDEXX population.

Age category	Odds ratio	95% confidence interval
0.0–3.0	0.26*	0.13–0.47
3.1–6.0	1.10	0.78–1.53
6.1–9.0	1.61*	1.20–2.16
9.1–12.0	1.27	0.93–1.73
12.1–15.0	0.98	0.65–1.48
15.1–18.0	0.38	0.10–1.55
18.1+	1.52	0.09–24.78

* , *p* < 0.050

The lymphoma population consisted of 56% males and 44% females, whereas the reference population consisted of 48% males and 52% females. The sex distribution for the two populations were significantly different and the lymphoma population had a significantly higher percentage of males (*χ*^2^: 4.55, *p* = 0.033). A significant relationship between sex (including neutering status) and population group (lymphoma vs. reference) (*χ*^2^: 10.97, *p* = 0.011) was found. Regarding neutering status, there was a significantly higher proportion of spayed females to intact females in the lymphoma population when compared with the reference population (*χ*^2^: 7.47, *p* = 0.006). The proportion of neutered males to intact males did not differ significantly between groups (*χ*^2^: 0.05, *p* = 0.800).

### Section Clinical Pathology database

A total of 43 cases were identified during the initial database search, of which four were excluded. Thirty-nine dogs with lymphoma were included from the CP database. The reference population consisted of 5569 dogs. The prevalence of lymphoma in this study population was 7.0 per 1000 dogs (0.70%).

The median age of dogs with lymphoma was 6.5 years (range: 2.0 months – 14.0 years), and the population consisted of 54% males and 46% females. It was not possible to extract age and sex data for the reference population; thus, no comparisons were possible.

From Table 2, two dog breeds had significantly increased odds for developing lymphoma compared with other breeds in this population. There were only three Springer Spaniels in the CP reference population; OR were not calculated for this breed.

### Differences between lymphoma study populations

No significant difference was found in terms of lymphoma prevalence for the two groups (*χ*^2^: 0.14, *p* = 0.700), and also between the age distributions of both groups (*p* = 0.456). When neutering status was compared between the two lymphoma groups, there was a higher proportion of both spayed females to intact females and neutered males to intact males in the CP population compared with the IDEXX lymphoma population (*χ*^2^: 12.01, *p* = 0.005 for females; *χ*^2^: 8.86, *p* = 0.003 for males).

## Ethical considerations

This was a retrospective study conducted on data obtained from clinical cases and ethical approval was not needed.

## Discussion

This study showed an increased prevalence of lymphoma in certain breeds in two South African study populations. The Labrador Retriever, Boerboel, Bull Terrier, Border Collie, Boxer, English Bulldog, Rhodesian Ridgeback, Basset Hound, Belgian Shepherd, Bullmastiff, Mastiff, Kerry Blue Terrier and Schipperke were identified from the IDEXX database to be at an increased risk for lymphoma. The CP study population showed an increased risk for lymphoma in the Belgian Shepherd and Newfoundland. Many of the breeds identified in the IDEXX database as having a significantly increased OR were also identified in the CP database as having OR > 1.00 but failed to reach statistical significance. The reason for this is likely to be the small CP study population size. Similarly, several other breeds in the IDEXX database had OR > 1.00 but failed to show statistical significance. This could be related to the fact that some breeds were represented by very few cases in this population.

Previous epidemiological studies have identified a number of breeds as having an increased risk for developing lymphoma. Among the breeds identified in this study, the Boxer is frequently described as being predisposed. Numerous studies conducted in North America (Dorn et al. 1967; Keller et al. 1993; Priester 1967; Priester & McKay 1980; Villamil et al. 2009), the United Kingdom (Day & Whitbread 1995; Edwards et al. 2003), France (Fournel-Fleury et al. 2002; Pastor et al. 2009; Ponce et al. 2010), the Netherlands (Teske 1994) and Italy (Gavazza et al. 2001) have shown this breed to be overrepresented when compared with a reference population. This breed also shows a predilection for T-cell lymphomas and lymphoproliferative disorders (Fournel-Fleury et al. 2002; Modiano et al. 2005; Pastor et al. 2009; Ponce et al. 2010), underscoring the role of genetics in the development of this disease. In contrast, two recent epidemiological studies in Germany (Ernst et al. 2016) and Switzerland (Gruentzig et al. 2016) failed to demonstrate a predilection for lymphoma in this breed.

In addition to the frequently cited Boxer, numerous studies have also identified the Bullmastiff (Day & Whitbread 1995; Edwards et al. 2003; Ernst et al. 2016; Keller et al. 1993; Villamil et al. 2009) and English Bulldog (Edwards et al. 2003; Priester & McKay 1980) as having a significantly higher relative risk for developing lymphoma, as was found here. In a case series following 59 Bullmastiffs from three households over a period of 3 years, 9 of these animals (some of which were related) developed lymphoma (Onions 1984). Based on these findings, it was thought that this breed had one of the highest incidence rates among dogs (Onions 1984). In a study evaluating the incidence of lymphoma in a population of insured dogs in the United Kingdom, a significant breed effect was demonstrated for all of the three above-mentioned breeds, and that certain age groups within each breed appeared to have an increased incidence of lymphoma compared with other age groups (Edwards et al. 2003). Specifically, a higher incidence of lymphoma was noted in the 0.0–3.0 year group for English Bulldogs, in the 4.0–6.0 year group for Bullmastiffs and in the 7.0–9.0 year and 10.0–14.0 year groups for Boxers. In our study, similar breed-specific analysis was not performed. However, it should be noted that most of the Bullmastiffs fell into the 4.0–6.0 year group, and most of the Boxers from this series were over 6 years of age. This suggests a similar pattern. It is also interesting to note that both the English Bulldog and the Boxer derive from the same ancestor, namely the Bullenbeisser (Bongianni & Mori 1982; Fogle 2000). The Bullmastiff in turn is derived from the English Bulldog and the English Mastiff (Bongianni & Mori 1982; Fogle 2000). All of these breeds fall into the Molosser category of dog breeds and are descended from the Tibetan Mastiff (Bongianni & Mori 1982). This further substantiates the role of genetics in the development of lymphoma and suggests that various susceptibility genes may have been inadvertently selected during derivation of these breeds.

Other breeds previously mentioned in the literature and identified in the current study as having increased odds of developing lymphoma include the Labrador Retriever (Priester & McKay 1980) and Basset Hound (Priester & McKay 1980; Villamil et al. 2009).

Although many of the results of the present study were in agreement with previous reports, some differences were also noted. Certain breeds have been mentioned in earlier studies as being overrepresented in lymphoma populations when compared with a reference population, but similar results were not found in our study. These breeds are listed in Table 4, together with the relevant studies. Of these breeds, the Rottweiler has featured in numerous studies, and familial clustering has been demonstrated in two case series, of which one series originated from South Africa (Lobetti 2009; Teske et al. 1994). However, the OR for this breed was not significantly greater than 1.00 in either of the South African study populations here. A possible explanation for this discrepancy could be that the clustering was observed in a family with a particular genetic susceptibility, which is not necessarily present in all South African Rottweilers.

**TABLE 4:** List of dog breeds that have been previously described in the veterinary literature as being overrepresented in lymphoma populations compared to their reference populations.

Dog breed	Reference(s)
Airedale Terrier	Priester and McKay (1980); Villamil et al. (2009)
American Pit Bull Terrier	Ernst et al. (2016)
American Staffordshire Terrier	Ernst et al. (2016)
Beagle	Keller et al. (1993); Priester and McKay (1980)
Bernese Mountain Dog	Villamil et al. (2009)
Bouvier des Flandres	Teske (1994); Villamil et al. (2009)
Briard	Ernst et al. (2016); Keller et al. (1993)
Cocker Spaniel	Pastor et al. (2009)
Collie	Day and Whitbread (1995)
Doberman	Day and Whitbread (1995)
German Shepherd	Day and Whitbread (1995)
Golden Retriever	Priester and McKay (1980); Villamil et al. (2009); Zemann et al. (1998)
Gordon Setter	Villamil et al. (2009)
Great Dane	Day and Whitbread (1995)
Hungarian Vizsla	Villamil et al. (2009)
Irish Setter	Ernst et al. (2016); Pastor et al. (2009)
Irish Water Spaniel	Keller et al. (1993)
Irish Wolfhound	Villamil et al. (2009)
Jack Russell Terrier	Day and Whitbread (1995)
Old English Sheepdog	Villamil et al. (2009)
Pembroke Welsh Corgi	Villamil et al. (2009)
Rottweiler	Day and Whitbread (1995); Ernst et al. (2016); Jagielski et al. (2002); Lobetti (2009); Teske (1994); Teske et al. (1994); Villamil et al. (2009)
Saint Bernard	Teske (1994); Villamil et al. (2009)
Scottish Terrier	Keller et al. (1993); Priester and McKay (1980); Teske (1994); Teske et al. (1994); Villamil et al. (2009)
Yorkshire Terrier	Villamil et al. (2009)

The relevant sources of the dog breed information have also been listed.

Note: Please see the full reference list of the article, Van Rooyen, L.J., Hooijberg, E. & Reyers, F., 2018, ‘Breed prevalence of canine lymphoma in South Africa’, *Journal of the South African Veterinary Association* 89(0), a1530. https://doi.org/10.4102/jsava.v89i0.1530, for more information.

Studies from Brazil (Neuwald et al. 2014), Poland (Sapierzynski et al. 2010), Romania (Cora et al. 2016), England and Scotland (Blackwood et al. 1997) mentioned that some of the above-mentioned breeds appeared to be overrepresented in terms of absolute numbers, but no statistical analysis was performed in terms of comparison with a reference population. An earlier study also stated that no breed predispositions were found when the lymphoma population was compared with numbers of dogs registered at the kennel clubs (Dobson & Gorman 1993). The details of the statistical analysis were unavailable; thus, no further comment can be made.

Another finding in the present study was that of a significantly decreased OR for the Maltese Poodle in the IDEXX population. A previous study found a significantly decreased OR for the Maltese breed, as well as a number of other breeds (Villamil et al. 2009). It should, however, be noted that the type of dog that is commonly referred to as a Maltese Poodle in South Africa is not a recognised breed (Z. Petersen [Kennel Union of South Africa], pers. comm., 27 February 2017). The Maltese, on the contrary, is a small white dog with a silky coat which belongs to the Toy group of dogs (Anonymous 2016). Thus, the findings of this study cannot be compared with those of the previous study and should be considered as unique to South Africa. Overall, the findings of decreased OR in several breeds suggest that various heritable protective factors exist, in addition to genetic risk factors (Modiano et al. 2005).

Several breeds with significantly increased risk for lymphoma in this study had not been mentioned previously in the literature. These included local breeds such as the Boerboel and Rhodesian Ridgeback, as well as the Border Collie, Bull Terrier, Belgian Shepherd, Newfoundland, Schipperke and Kerry Blue Terrier. The latter three breeds appeared to have markedly increased OR; however, these breeds were relatively underrepresented in both reference populations, and the small sample size is evidenced by the very wide CI. Border Collies were found in a previous study to have a predilection for B lymphocyte lymphoproliferative disorders (Modiano et al. 2005).

Although the ancestry of the Boerboel is not entirely clear, available records suggest that this breed was derived from local dogs and Molosser-type dogs which were imported by early European settlers, namely the Bullenbeisser, English Bulldog, English Mastiff and eventually the Bullmastiff (Graebe 1995). Similarly, it is thought that the Rhodesian Ridgeback derives from Dutch and German Mastiffs crossed with indigenous ridgebacked dogs (Fogle 2000). These findings again suggest the existence of susceptibility genes which may be common to a subset of Molosser-type dogs.

There are a number of potential reasons for the discrepancies found between the results of this study and those of previous findings. Firstly, some of the dog breeds which have been mentioned in the literature as having an increased risk for lymphoma but did not feature in this study are not particularly popular in South Africa. This is evidenced by the low frequency of these breeds in the various reference populations used for this study, as well as in the insurance database. Some examples of such breeds include the Airedale Terrier, Old English Sheepdog, Briard, Bernese Mountain Dog and Irish Water Spaniel. With very low numbers in the South African general canine population, such disease predispositions and susceptibilities cannot be easily demonstrated.

Secondly, the composition of the reference populations used differ from study to study. In this study, the two reference populations used for the purpose of comparison included canine patients presenting for veterinary care and undergoing clinicopathologic or microbiologic testing. While using a hospital population as a reference population is commonly employed in veterinary studies (Ernst et al. 2016), this approach may have drawbacks. Some populations may only comprise sick animals and may not be representative of the general canine population (Ernst et al. 2016). On the contrary, while the use of an insurance population may be more representative of a healthy population, it may bias the reference population towards more expensive breeds (Ernst et al. 2016).

Another possibility for the different findings include genotypic differences between dogs of the same breed in different geographic locations (Dobson 2013), and that overrepresented breeds in one location are specific to that country (e.g. the Boerboel in South Africa) (Pastor et al. 2009). Depending on population size and breed popularity, some breeds may have a great deal of genetic diversity across geographical locations (Dobson 2013). This is speculated to be the reason for the higher incidence of haemangiosarcoma in American Golden Retrievers compared with British Golden Retrievers (Dobson 2013), for example. This could also partly account for the differences in progression-free survival of dogs with lymphoma from different geographical locations, as was shown by Wilson-Robles et al. (2017). In terms of lymphoma prevalence, it is possible that a similar situation exists for the South African variants of certain breeds compared with their counterparts found in other locations. Furthermore, differences between early and more recent studies could reflect evolution of the various dog breeds over time, with a resultant change in the incidence of lymphoma (Pastor et al. 2009). This could explain the difference in some of the findings between the present study and those studies conducted decades ago.

The aetiology of lymphoma is considered to be multifactorial, with both environmental and genetic influences implicated in both humans and dogs (Marconato, Gelain & Comazzi 2013; Modiano et al. 2005; Pastor et al. 2009). Cancer is a genetic disease associated with the accumulation of mutations and rearrangement of DNA (Thamm et al. 2013). Various insults produce oncogenic mutations, which may give rise to a neoplastic phenotype (Thamm et al. 2013).

Heritability plays a key role in the development of spontaneous lymphoproliferative disease (Modiano et al. 2005; Thamm et al. 2013), as evidenced by the fact that certain breeds have a specific predisposition to different subtypes (Modiano et al. 2005). Moreover, the finding that some breeds appear to be predisposed to lymphoma, while others appear underrepresented, suggests that genetic risk or protective factors for the disease were segregated with breed-specific traits and perpetuated with inbreeding and line breeding (Modiano et al. 2005). This is supported by the findings from the present study, which demonstrates that a number of related breeds all appear to have an increased risk for lymphoma.

Different cytogenetic abnormalities and mutations have already been identified in dogs with lymphoproliferative disease, and recurrent abnormalities may occur with significantly higher frequency in a single dog breed (Modiano et al. 2005). Epigenetic modifications, such as genomic hypomethylation, are also implicated in the development of spontaneous lymphoproliferative disease in dogs and humans (Pelham et al. 2003). Interestingly, in a previous study, evidence of hypomethylation was found in only one-third of lymphoid leukaemia cases and two-thirds of lymphoma cases, reinforcing the hypothesis that there are multiple molecular genetic pathways which could result in the neoplastic phenotype (Pelham et al. 2003). It is possible that a number of these genetic and epigenetic abnormalities may have been responsible for the breed predispositions found in this study.

The role of environmental agents has also been studied. Specifically, associations between the development of non-Hodgkin’s lymphoma and exposure to herbicides (Hayes et al. 1991), electromagnetic radiation (Reif, Lower & Ogilvie 1995), living in industrial areas (Gavazza et al. 2001) and the use of paints and solvents by dog owners (Gavazza et al. 2001) have been described. A French study also demonstrated a significant association between the distribution of canine non-Hodgkin’s lymphoma and that of waste incinerators, polluted sites and radioactive waste (Pastor et al. 2009). In the current study, the case histories were mostly unknown to the investigators. Thus, it was not possible to ascertain whether any of these factors contributed to the development of lymphoma in the cases included in this study.

In terms of the age data, the median age for the CP lymphoma population was 6.5 years (mean 7.3 years), whereas the median age of the IDEXX lymphoma population was 8.0 years (mean 7.7 years). These findings are in agreement with previous studies reporting median and average ages (Blackwood et al. 1997; Cora et al. 2016; Day & Whitbread 1995; Dobson & Gorman 1993; Dorn et al. 1967; Ernst et al. 2016; Fournel-Fleury et al. 2002; Greenlee et al. 1990; Gruentzig et al. 2016; Jagielski et al. 2002; Keller et al. 1993; Neuwald et al. 2014; Pastor et al. 2009; Ponce et al. 2010; Zemann et al. 1998). The different age distribution of the IDEXX reference population compared with the IDEXX lymphoma population could be because of increased numbers of animals with lymphoma in the 6.1–9.0 year bracket, as described below.

The significantly decreased OR for the 0.0–3.0 year group has also been described by Villamil et al. (2009). A similar situation exists in humans, where children have a very low incidence of non-Hodgkin’s lymphoma (Fisher & Fisher 2004). Given that numerous environmental agents may play a role in mutagenesis and lymphoma pathogenesis, it is possible that the low incidence of lymphoma in the young of both species could be because of limited exposure to these mutagens during a short lifetime. The finding of an increased OR for the 6.1–9.0 year group in the IDEXX lymphoma population is also in agreement with the peak age brackets reported by other studies (Cora et al. 2016; Day & Whitbread 1995; Dobson & Gorman 1993; Ernst et al. 2016; Gavazza et al. 2001; Gruentzig et al. 2015; Merlo et al. 2008; Neuwald et al. 2014; Teske 1994; Villamil et al. 2009). These ranges differed slightly from study to study (most likely because of different age structures of the various populations) but generally included the late middle age to early geriatric group. The oldest peak age reported was 10.0 years (Edwards et al. 2003). In one study, the highest incidence rate for canine lymphoma was reported to be in the 7.0–9.0 year group, which was in contrast to the peak ages for other neoplastic disorders (9.0–11.0 years) (Merlo et al. 2008). No particular reason was offered for this discrepancy.

The impact of sex and neutering on the development of canine lymphoma has been a contentious issue in the veterinary literature. Many previous studies did not compare their sex and neutering prevalence data with that of a reference population, which limits the usefulness of their findings. While some studies have reported that sex and neutering status do not play a role in the development of lymphoma (Dorn et al. 1967; Ernst et al. 2016; Fournel-Fleury et al. 2002; Gavazza et al. 2001; Greenlee et al. 1990; Merlo et al. 2008; Modiano et al. 2005; Ponce et al. 2010; Teske 1994), the current study showed that the IDEXX lymphoma population had a significantly increased number of males and neutered females compared with the reference population. These findings are in agreement with the study by Villamil et al. (2009), which found that intact females had a significantly lower risk for lymphoma. Other studies have also found similar results (Gruentzig et al. 2016; Keller et al. 1993; Priester & McKay 1980). In fact, two of these studies demonstrated that in addition to neutered females, neutered males were at a greater risk of developing lymphoma (Gruentzig et al. 2016; Keller et al. 1993), which was not found in this study. This has also been noted in breed-specific studies conducted in Golden Retrievers (Riva et al. 2013) and Hungarian Vizslas (Zink et al. 2014). In humans, women have a lower incidence of lymphoma compared with men, with the difference in incidence decreasing after the age of 50 (age commonly associated with menopause) (Villamil et al. 2009). It is thought that the interaction of sex hormones with oestrogen or progesterone receptors influences gene expression (Villamil et al. 2009). A similar mechanism may exist in dogs.

The finding of the increased proportions of sterilised animals in the CP lymphoma population compared with the IDEXX lymphoma population could be because of the format of the submission forms used by each laboratory, as no option for ‘neutering status’ is present on the IDEXX submission form, and it is often not disclosed. Although this could influence the accuracy of our findings regarding the sex and neutering effect in the IDEXX population, the large sample sizes provide some robustness (i.e. the same pattern probably occurs uniformly when completing forms for dogs with and without lymphoma).

The overall prevalence of lymphoma in the two study populations did not differ significantly from each other. These findings are similar to the prevalence calculated from the German (0.78%) (Ernst et al. 2016) and Polish (0.81%) (Jagielski et al. 2002) studies, with an own clinic population used as reference population in both instances. Other estimates of prevalence in the literature include 0.08% (Edwards et al. 2003), 0.28% (Priester & McKay 1980), 0.58% (Keller et al. 1993), 1.20% (Villamil et al. 2009), 2.00% (Gruentzig et al. 2016) and 2.59% (Cora et al. 2016). The prevalence may vary depending on the composition of the populations under study and the techniques used to diagnose lymphoma. For example, the latter two studies consisted of cases from necropsy studies as well as cytological and histopathological studies, which would skew the results. Comprehensive necropsy examination may have provided a more sensitive means to ultimately diagnose lymphoma compared with cytological or histopathological examination of a limited number of tissues.

## Limitations of the study

This study had several limitations. Small sample numbers could have masked significant findings. The reference populations consisted of a large number of sick animals, which might not be truly representative of the South African dog population. As a retrospective analysis, the findings are only as accurate as the data recording. Very few cases were confirmed histopathologically or with immunophenotyping. In many cases, a comprehensive history was lacking.

## Conclusion

Numerous breeds in this study, such as the Boxer, Boerboel, Rhodesian Ridgeback, English Bulldog and Bullmastiff, showed significantly increased odds for developing lymphoma compared with other breeds. This may be because of the genotypic differences that are specific to South African variants of these breeds. As for previous studies, the age of onset of canine lymphoma appears to be lower than that of other neoplastic diseases. The findings regarding the impact of sex and neutering status suggest that reproductive hormones play a role in the development of this disease. Practically, these findings may be useful to veterinarians advising pet owners regarding potential diseases which may occur in their pet, and also in terms of the pros and cons of neutering animals. The findings may allow clinicians to be more vigilant in terms of monitoring certain breeds for the development of lymphoma. This study may also serve as a baseline for future research investigating the genetic mechanisms involved in the development of lymphoma.
